# Clinical learning environment for nursing students in operating rooms: Development of an evaluation scale

**DOI:** 10.1016/j.heliyon.2024.e24553

**Published:** 2024-01-24

**Authors:** Rui Zhang, Yuqi Wu, Li li

**Affiliations:** a Operating Room, The First Hospital of China Medical University, Shenyang, 110001, China; b The First Hospital of China Medical University, No.155, Nanjing North Street, Heping District, Shenyang, China

**Keywords:** Nursing students, Operating rooms, Clinical learning environment, Scale development

## Abstract

**Aim:**

To develop a measurement tool to evaluate the clinical learning environment for nursing students in operating rooms.

**Background:**

In this study, a scale for evaluating the clinical learning environment for nursing students in operating rooms was developed and subjected to reliability and validity tests.

**Design:**

A cross-sectional, methodological study.

**Methods:**

Qualitative interviews, the Delphi method, a literature review and pilot testing were employed to develop the scale. A purposive sampling method was used to select September 2021 through May 2022; a total of 227 nursing students with internship experience in operating rooms at several teaching hospitals in North China were selected to evaluate the reliability and validity of the scale.

**Results:**

The 32-item, four-dimensional evaluation scale was developed through two rounds of consultation with 17 experts. The reliability and validity test showed that the overall Cronbach's alpha was 0.984 and 0.96. The split-half reliability for the total scale was 0.937, indicating good reliability.

**Conclusion:**

The proposed scale has high reliability and validity in evaluating the clinical learning environment of nursing students in operating rooms and improving clinical nursing education.

## 1 Introduction

Nursing places emphasis on the acquisition of practical skills. Clinical internship, one of the primary methods of practical nursing education, influences nursing students' transition from theoretical to practical education [1]. Nursing students’ development of professional values and advancement of professionalism during the internship process is directly tied to the clinical learning experience, in which the clinical learning environment plays a significant role. According to Dunns and other academics [2], the clinical learning environment is similar to a tangled web that includes every element affecting students' learning ability. On the one hand, the clinical learning environment includes diverse and complicated external influences; on the other hand, it also includes measurable and internal factors that the nursing students themselves impact.

According to research by Meyer and others [3], nursing students see the operating room as a setting where surgical nurses play a crucial part in a situation that calls for quick thinking. Nursing students can study aseptic procedures, such as surgical hand cleaning, donning and donning sterile gowns, and passing sterile equipment in the operating room that is less frequently covered on the wards. In addition to providing specialized clinical training and development for nursing students in anesthesia, surgical settings, and surgical positions, the operating room also aids in their understanding of anatomy, physiology, pharmacology, pain management, and the majority of common diseases [4]. Therefore, the operating room is a superior setting for nursing students to quickly develop their general clinical skills in surgery and their clinical problem-solving skills.

Most national and international studies on clinical learning environments have focused on the overall internship environment [5]. Concerning operating room nursing students, the relevant studies mainly tackle the innovation of teaching methods and the impact of students' psychological factors on the effectiveness of internships ^[^ [6,7]^]^. Overall, nursing students expressed moderate agreement and satisfaction with the clinical learning environment. However, there are several issues, including a lack of individualization, weak innovativeness, unclear task orientation and ambiguous roles of the teaching faculty. These contradictions become more noticeable when entering the operating room. Little attention has been paid to evaluating the operating rooms' clinical learning environment. Educators and managers of operating room nursing need to focus on evaluating and improving the environment to enhance the effectiveness and experience of students’ internships ^[^ [3,8]^]^. However, few specific evaluation tools exist for the unique operating room environment, locally and globally [9]. Urgent action is required to develop a rigorous and appropriate instrument for students to evaluate the clinical learning environment in operating rooms.

Consequently, a customized assessment tool has to be developed and validated among nursing students to provide objective evidence for assessing the clinical learning environment in operating rooms. Therefore, this study aimed to develop a reliable and valid tool through a literature review, qualitative interviews and expert consultations to enable operating room nursing students to evaluate the clinical learning environment.

## 2 Methods

### 2.1 Research design

The research process included a literature review, program design, review writing, scale construction, data collection, scale validation, semi-structured interview guide development and qualitative interviews.

Two rounds of expert consultation utilizing the Delphi method to modify, eliminate and add relevant items. The items questioned by the experts had to be clarified or altered to form the initialized scale for the clinical learning environment of the nursing students in the operating room. Then, item analysis, reliability analysis, and validity tests were done to formulate the evaluation scale for the clinical environment of nursing students in the operating room.

### 2.2 Initialization of the item pool

The relevant foreign and domestic literature on “clinical learning environment,” “nursing students,” and “operating room internship” was consulted to clarify the purpose and objectives of the scale. On this basis, a discussion was held among our research team members to set up the connotation framework of the clinical learning environment in operating rooms, determine the scale's dimensions, and initialize the item pool.

### 2.3 Qualitative research

#### 2.3.1 Sampling method and determination of sample size

Using purposive sampling, clinical educators and operating room nursing students from Level A tertiary hospitals in Liaoning Province were recruited for semi-structured interviews between September 2021 and May 2022.

The selection of interviewees was predicated upon reaching the point of information saturation, shereby the number of available participants would suffice. Consequently, the research team determined that the final sample size for the interviews would consist of five members from the clinical teaching faculty and fifteen nursing interns in the operationg room.

#### 2.3.2 Inclusion criteria

Inclusion criteria for nursing students: (1) full-time nursing undergraduates; (2) internship duration in operating rooms ≥3 weeks; (3) internship in a Level A tertiary teaching hospital; (4) voluntary participation and the ability to express true feelings about the internship.

Inclusion criteria for clinical educators: (1) responsible for nursing internship teaching in operating rooms; (2) teaching experience in this field ≥1 year; (3) working in a Level A tertiary teaching hospital; and (4) voluntary participation in this study.

#### 2.3.3 Interview outline

The interview outline was used as a guide for the discussion and was modified as needed during the interview.

Nursing student interview outline: ① What do you think of the clinical internship environment in the operating room? ② What difficulties have you encountered during your internship in the operating room? ③ How do you get along with the teaching staff and other colleagues during your internship?

Outline of the interview with the teaching faculty? ① What do you think of the clinical practice environment in the operating room? ② What difficulties have you encountered in teaching in the operating room? ③ How do you get along with the nursing interns in the teaching process?

### 2.4 Delphi expert consultation

#### 2.4.1 Expert selection

A total of 17 authoritative experts in operating room nursing management, clinical nursing in operating rooms, and nursing education were invited [10]. The inclusion criteria are as follows: (1) undergraduate degree or above; (2) intermediate professional title or above; (3) specialized nurses engaged in clinical nursing in operating rooms or operating room nursing management for more than 15 years; (4) a rigorous research attitude and voluntary participation in this study; (5) ability to fully participate in the consultation process.

#### 2.4.2 Expert consultation

##### 2.4.2.1 First round of expert consultation

The questionnaire includes (1) a survey of the experts’ fundamental knowledge and (2) a questionnaire regarding the clinical learning environment evaluation scale for operating room nursing students.

Based on their own practical experience and professional knowledge in this study, experts evaluated the judgment basis of the indices Ca and their familiarity with the indices *Cs*. Then, the specific values using the authoritative coefficient formula *Cr*=(*Ca* + *Cs*)*2 were calculated.

##### 2.4.2.2 Second round of expert consultation

The experts' opinions on the first round of consultation were summarized, organized, and analyzed. Based on their opinions, items were added, deleted, modified, and then combined based on their feedback. Within a week, the questionnaire was sent to the experts with reminders to respond promptly. After the second round, the questionnaire's content was adjusted according to the experts' opinions. Due to the increased focus on expert opinions in this round, the consultation process was terminated, and the clinical learning environment evaluation scale for operating room nursing students was developed.

#### 2.4.3 Statistical analysis

After collecting all the responses, a Microsoft Excel database was established, and the relevant data were processed and analyzed using SPSS 26.0. The mean and standard deviation of each item and each dimension were collected, plus the general information of the experts. The experts’ positivity, opinion coordination, and authority degrees were evaluated.

**Fig. 1:**
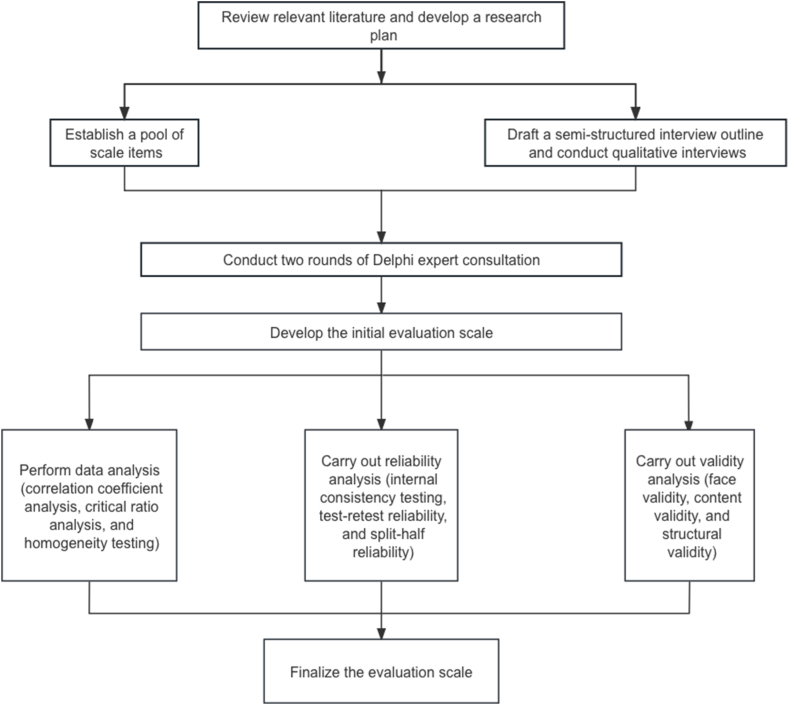
The framework of the scale development.

This study proposed the framework for developing the evaluation scale for the clinical learning environment of operating room nursing students based on literature research and qualitative interviews, as shown in Fig. 1.

### 2.5 Reliability and validity tests

#### 2.5.1 Survey objects

Between September 2021 and May 2022, 227 nursing students who had completed an operating room internship at a Level A tertiary teaching hospital were selected using a convenience sampling method. The ethics committee of the First Hospital of China Medical University approved this study. The inclusion criteria were as follows.

(1) The internship took place in a Level A tertiary teaching hospital.
(2) The internship period lasted from September 2021 to May 2022.
(3) Completion of 6–8 clinical units during the one-year internship period, and the internship in operating rooms was ≥ three weeks;
(4) The participants provided informed consent and were willing to participate in the study.

#### 2.5.2 Research tools

2.5.2.1 A self-designed survey questionnaire was prepared to collect general information about the nursing students, such as gender, education level, time spent in operating rooms during the internship, duration of the operating room internship and the name of the hospital where the internship was completed.

Evaluation questionnaire.

2.5.2.2. The internship experience of the nursing students in operating rooms was surveyed using the proposed evaluation scale of the clinical learning environment for operating room nursing students. The scale consists of 36 items in five dimensions. A 5-point Liikert scale was utilized (1 = strongly disagree, 2 = disagree, 3 = undecided, 4 = agree, 5 = strongly agree), with the total score ranging from 0 to 180. Higher scores indicate a better evaluation of the clinical learning environment in operating rooms.

#### 2.5.3 Statistical analysis

After the responses were collected, a Microsoft Excel database was established, and the data were processed and analyzed using SPSS 26.0. Two researchers independently entered the data to ensure accuracy.

##### 2.5.3.1 Reliability testing

(1) Internal consistency testing

Cronbach's alpha and split-half reliability were calculated for each dimension and the overall scale.

(2) Test–retest reliability

Two weeks later, 40 nursing students who had participated in the initial survey were re-interviewed using the same questionnaire, and the correlation between the two surveys was assessed.

##### 2.5.3.2 Validity testing

(1) Surface Validity

Nurse trainees in the operating room were invited to complete the questionnaire, checked for coherent and clear verbal and semantic expression, and calculated the length of time taken to complete the questionnaire.

(2) Content Validity

Experts evaluated the content validity of each item, and the item content validity index (I-CVI) and the scale content validity index (S-CVI) were calculated.

(3) Structural Validity

Exploratory factor analysis (EFA) was used to evaluate structural validity.

## 3 Results

### 3.1 Initialization of the item pool

The item pool for the clinical learning environment evaluation scale for operating room nursing students was initiated through literature research. The initial sample consisted of 65 items distributed across four dimensions: work environment (15 items), learning environment (15 items), interpersonal communication (20 items), and operating room teaching and practice (15 items).

### 3.2 Qualitative findings

#### 3.2.1 Interview results

Twenty in-depth, one-on-one interviews were conducted, and after coding, excerpting, and interpreting their content, five themes emerged: work environment, interpersonal relationships, clinical practice, task orientation, and personality and innovation.

#### 3.2.2 Formation of the first round of expert questionnaires

Through the interviews with operating room interns and preceptors and repeated discussions within the research team, a scale consisting of 5 dimensions and 42 items was developed, including work environment (8 items), interpersonal relationship (8 items), clinical practice (9 items), task positioning (9 items), and individuality and innovation (8 items).

### 3.3 Delphi expert consultation results

In the second round, 17 experts were consulted. The experts participating in each round were highly representative and authoritative. They come from the professional fields of operating room clinical nursing, operating room nursing management, and nursing education.

#### 3.3.1 Positive and authoritative degrees of expert

The authoritative coefficients for the two rounds of consultation in this study were 0.92 and 0.93, respectively, indicating that the authority of the selected experts is reliable and that the consultation results are highly credible (Table 1).

**Table 1:** Authoritative coefficients of experts (n = 17).

Consultation round	Judgment basis of the indices (Ca)	Familiarity with the indices (Cs)	Authoritative coefficient (Cr)
First round	0.96	0.87	0.92
Second round	0.95	0.92	0.93

#### 3.3.2 Concentration and coordination of expert opinions

In the first round of consultation, the CV ranged from 0.049 to 0.212, and the item CV ranged from 0.049 to 0.438. In the second round of consultation, item CV varied from 0 to 0.23. Kendall's harmony coefficient of items stood at 0.115. The P-value of the test was less than 0.01, indicating good coordination of expert opinions (Table 2).

**Table 2:** Coordination of expert opinions.

Consultation round	CV	Kendall's harmony coefficient (CS)	Mean importance score
First round			
Dimension	0.049–0.212	0.143	4.41–4.94
Item	0.049–0.438	0.141	3.53–4.94
Second round			
Item	0–0.23	0.115	4.41–5

#### 3.3.3 Expert opinions and scale modifications

**Fig. 2:**
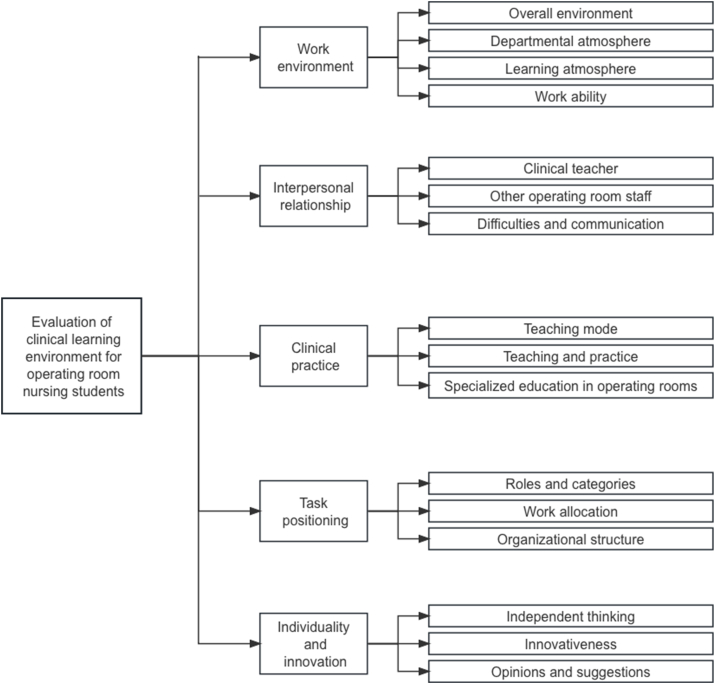
Technology roadmap.

Fig. 2 summarizes the design process of the evaluation scale. After the first round of expert consultation, 36 questionnaire items were submitted for the second round. After the second round of expert consultation, four items were modified based on screening criteria and expert textual opinions, resulting in an initial scale of 36 items in five dimensions.

### 3.4 Reliability and validity test results

#### 3.4.1 Reliability test of our scale

##### 3.4.1.1 Internal consistency test

Cronbach's alpha and split-half reliability were calculated for each dimension and the overall scale. The results indicate that Cronbach's alpha ranged from 0.948 to 0.967 for each dimension, with an overall value of 0.984. The split-half reliability for each dimension ranged from 0.882 to 0.951, and the overall split-half reliability was 0.937.

##### 3.4.1.2 Test-retest reliability

Forty nursing students were polled a second time two weeks after completing the initial questionnaire, and 40 valid responses were received. The results indicate that the test-retest reliability of each dimension was between 0.762 and 0.782, while the test-retest reliability of the overall scale was 0.788. See Table 3 for details.

**Table 3:** Cronbach's alpha, split-half reliability, and test-retest reliability.

Dimensions	Cronbach's alpha	Split-half reliability	Test-retest reliability
Work environment	0.951	0.892	0.769
Interpersonal relationship	0.967	0.951	0.762
Clinical practice	0.946	0.882	0.771
Task orientation	0.948	0.885	0.751
Individuality and innovation	0.954	0.930	0.782
Overall scale	0.984	0.937	0.788

#### 3.4.2 Validity testing of our scale

##### 3.4.2.1 Face validity

At the commencement of the survey, an invitation was extended to five nursing interns to complete the pretest scale. They all provided feedback indicating that the questionnaire's content was comprehensible and devoid of ambiguity. Furthermore, after an extensive examination of national and international literature, the researcher developed a preliminary pretest scale for assessing the clinical learning environment in the operating room. This was achieved by utilizing the outcomes of semi-structured interviews and Delphi expert correspondence and employing various statistical methods to scrutinize the entries. As a result, the pretest scale demonstrated favorable face validity.

##### 3.4.2.2 Content validity

The calculation of content validity probes whether the content of the entries in a measurement instrument meets the requirements of the concepts to be measured and is often judged by expert evaluators. In this study, five experts were invited to evaluate the content validity of the developed scale [11]. There are four scoring levels: 1 is irrelevant, 2 is weakly relevant, 3 is more relevant and 4 is very relevant. Based on the expert ratings, the I-CVI and the S-CVI were calculated. The results showed that the I-CVI of each item on the scale ranged from 0.90 to 1.00, and the S-CVI of the overall scale was 0.96.

##### 3.4.2.3 Structural validity

Structural validity is frequently employed to determine whether a scale conforms to the anticipated structure. It is a standard approach to scale development. The Kaiser–Meyer–Olkin (KMO) value was adopted during EFA to assess suitability for factor analysis. A KMO above 0.7 was considered suitable for factor analysis.

(1) The first EPA

The results show that the KMO value of the pilot scale was 0.955, and the result of Bartlett's test of sphericity reached a significant level (χ^2^ = 10127.509, *p* < 0.01), indicating that the study is very suitable for factor analysis (Table 4). Meanwhile, the scree plot shows that the slope flattened after the fourth factor. After a brief discussion, the research team determined that the four common factors were better than the original pilot scale with five dimensions. Without changing the work environment and interpersonal relationship dimensions, the team merged and revised three other dimensions, namely clinical practice, task orientation, and individuality and innovation, into two dimensions, namely, task orientation and practice and evaluation.

(2) The second EPA

**Table 4:** Variance explained by the four common factors in the first-factor analysis.

Factors	Initial eigenvalues			Extraction sums of squared loadings			Rotation sums of squared loadings		
	Total	% of variance	Cumulative %	Total	% of variance	Cumulative %	Total	% of variance	Cumulative %
1	23.365	64.903	64.903	23.365	64.903	64.903	9.334	25.929	25.929
2	2.017	5.601	70.504	2.017	5.601	70.504	6.738	18.716	44.645
3	1.394	3.872	74.376	1.394	3.872	74.376	6.414	17.817	62.462
4	1.083	3.009	77.386	1.083	3.009	77.386	5.372	14.923	77.386

Bartlett's sphericity test results showed that the KMO value amounted to 0.959, reaching the significant Level (χ^2^ = 8777.45, *p* < 0.01). The common factors and the items in Table 5 dominated by each factor were matched with the modified content structure of the scale, indicating the scale's structural validity. (Table 5, Table 6).

**Table 5:** Variance explained by the four common factors in the second-factor analysis.

Factors	Initial eigenvalues			Extraction sums of squared loadings			Rotation sums of squared loadings		
	Total	% of variance	Cumulative %	Total	% of variance	Cumulative %	Total	% of variance	Cumulative %
1	20.667	64.583	64.583	20.667	64.583	64.583	8.168	25.524	25.524
2	1.994	6.232	70.815	1.994	6.232	70.815	5.965	18.640	44.164
3	1.385	4.328	75.143	1.385	4.328	75.143	5.884	18.386	62.550
4	1.077	3.365	78.508	1.077	3.365	78.508	5.107	15.958	78.508

**Table 6:** Rotated component matrix of the second-factor analysis.

Dimension	Item	Factor 1	Factor 2	Factor 3	Factor 4
Practice and evaluation	35	0.784			
	32	0.782			
	33	0.772			
	34	0.763			
	30	0.755			
	31	0.749			
	36	0.745			
	29	0.701			
	27	0.686			
	28	0.684			
	26	0.569			
Task orientation	21		0.776		
	19		0.728		
	20		0.716		
	22		0.679		
	18		0.644		
	24		0.658		
	23		0.644		
	25		0.547		
Work environment	1			0.790	
	2			0.769	
	4			0.752	
	3			0.743	
	6			0.726	
	7			0.678	
	5			0.650	
Interpersonal relationship	10				0.748
	9				0.739
	8				0.734
	11				0.706
	13				0.703
	12				0.698

## 4 Discussion

Internships in clinical settings are an integral aspect of nursing education. The environment of clinical internships is crucial to the learning and development of nursing students. Operating rooms differ from ordinary wards in terms of the environment, management mode, professional knowledge, and internship requirements. Targeted evaluation of the clinical learning environment in operating rooms is required to establish a foundation for the environment's continuous optimization. By focusing on areas where improvement is most needed, the evaluation process can assist nurse managers and students to make the most of their time in the operating room to improve learning outcomes [12].

Reference has been made to relevant international learning environment evaluation scales, such as the Clinical Learning Environment Inventory (CLEI) [13], Clinical Learning Environment and Supervision Scale (CLES) [14], in the design concept and content of this scale to clarify the scope of the evaluation of the clinical learning environment in the operating room. Unlike the clinical learning environment evaluation scales widely used internationally, the present scale focuses on the nursing education environment in China. We explored the understanding and experience of nursing students and clinical instructors about the clinical learning environment in the operating room. The scale focuses on the local nursing education environment. On this basis, we combined the operating room's clinical learning requirements to make the scale's content more appropriate for the clinical learning requirements at this stage. In addition, due to the differences between China and the West in terms of content expression, there are more abstract evaluation descriptions on foreign scales.

In contrast, this scale is more inclined to practical contextualized expressions, such as “admission training”, “participation in practicing nursing care for various types of simple first- and second-level surgeries”, “specialized nursing care for operating room nurses”, “nursing care for the operating room nurses”, “nursing care for the operating room nurses”, “nursing care for the operating room nurses”, and “nursing care for the operating room nurses”. This scale is more oriented to practical contextualized expressions, such as “admission training”, “participation in practicing a variety of simple types of first- and second-degree surgical care”, and “specialized training for operating room nurses”. This might enable nursing students to evaluate the learning environment more accurately in a fixed context. The scales of previous studies contained six dimensions, and this study categorized the clinical learning environment in the operating room into four dimensions: work environment, interpersonal relationships, task orientation and practice and evaluation.

The scale designed in this study provides a method from the perspective of nursing students to discover problems in the clinical learning environment. It divides the clinical learning environment of operating rooms into four dimensions: work environment, interpersonal relationship, task orientation and practice and evaluation. Experts believe that these four dimensions can comprehensively and accurately evaluate the learning environment of operating room nursing students. Apart from evaluating essential elements, the proposed scale includes particular elements unique to operating rooms, such as the working environment, surgical cooperation and teaching methods. Overall, the scale is in line with current clinical practice.

Reliability was evaluated by internal consistency and test-retest reliability, aiming to determine whether the results measured by the proposed scale were consistent and stable [15]. The overall Cronbach's alpha was 0.984, and the overall split-half reliability was 0.937. Stability was represented by test–retest reliability. The overall test-retest reliability of the proposed evaluation scale for the clinical learning environment of operating room nursing students was 0.788 %, indicating its high reliability. Consequently, the proposed scale is consistent and stable.

Validity was evaluated to reflect whether the proposed scale was accurate and effective. The higher the validity, the more reliable the evaluation results and the higher the fitness between the results and the evaluated content [16]. Here, content validity is evaluated using I-CVI and S-CVI. The I-CVI of each item ranged from 0.90 to 1.00, and the S-CVI of the scale was 0.96. In addition, structural validity was tested through the EPA. After two EPAs, the content of the scale was further refined, suggesting that the items fit with the concepts reflected by the clinical learning environment in operating rooms and that the scale was valid.

This study combines qualitative and quantitative research methods to develop a preliminary evaluation scale for the clinical learning environment of operating room nursing students. The proposed scale includes 32 items in four dimensions, with a total score range of 32–160 points. The higher the overall score, the better the nursing students evaluate the clinical learning environment in operating rooms.

The research has practical implications for improving the clinical learning environment, practical teaching ability, and internship efficiency in operating rooms. In the future, more subjects will be surveyed, and the reliability and validity of the scale will be further tested to improve its quality.

## 5 Conclusion

In this study, the clinical learning environment evaluation scale for nursing students in operating rooms was developed according to the principles of scale development, with four dimensions and 32 entries, namely, work environment (7 entries), interpersonal relationship (6 entries), task orientation (8 entries), and practice and evaluation (11 entries). This scale provides a reliable, valid, and comprehensive evaluation of the clinical learning environment of nursing students in operating rooms. It can be applied to understand nursing students’ evaluation of the clinical learning environment and to improve clinical nursing teaching in operating rooms.

This study has several limitations. At the reliability testing stage, only samples from northern China multi-center hospitals were used, which limited the representativeness of nursing students in operating room internships. The small sample size and lack of validation factor analysis were due to workforce and time constraints. Future studies should expand the source and number of samples to ensure scientific and reliable content. The scale's validity was not tested for correlational validity in this study because there is no scale for the clinical learning environment of nursing in the operating room in China.

## Ethics statement

This study was conducted in accordance with the ethical standards of the Declaration of Helsinki, and the institutional ethics review board of our hospital approved the study protocol (approval number #[2021]449).

## Funding

This research did not receive any specific grant from funding agencies in the public, commercial, or not-for-profit sectors.

## Data availability statement

Data included in article/referenced in article. Data will be made available on request.

## Additional information

NO additional information is available for this paper.

## CRediT authorship contribution statement

**Rui Zhang:** Writing – review & editing. **Yuqi Wu:** Writing – review & editing, Writing – original draft, Visualization, Validation, Supervision, Software, Resources, Project administration, Formal analysis. **Li li:** Project administration, Methodology, Investigation, Formal analysis, Data curation.

## Declaration of competing interest

The authors declare that they have no known competing financial interests or personal relationships that could have appeared to influence the work reported in this paper.

## Figures and Tables

**Table:** 

	完全不同意	比较不同意	不确定	比较同意	完全同意
1 手术室有良好的工作氛围	○	○	○	○	○
2 手术室有良好的学习氛围	○	○	○	○	○
3 手术室工作人员之间相处融洽	○	○	○	○	○
4 手术室工作人员手术配合默契	○	○	○	○	○
5 手术室有详细的入科介绍和培训,帮助护生尽快适应新环境	○	○	○	○	○
6 手术室可以保障护生更衣和用餐等生活条件	○	○	○	○	○
7 手术室能为护生提供良好的学习环境,如示教室、教学设备等	○	○	○	○	○
1 临床带教教师及其他工作人员尊重护生,礼貌待人	○	○	○	○	○
2 临床带教教师能耐心示教,解答护生提出的问题	○	○	○	○	○
3 临床带教教师及其他工作人员注意言传身教,为护生做出榜样	○	○	○	○	○
4 临床带教教师与护生关系融洽,交流顺畅,掌握护生的实习需要	○	○	○	○	○
5 临床带教教师团队给护生提供支持和帮助	○	○	○	○	○
6 手术室临床实习负责人能经常与护生交流,了解实习情况	○	○	○	○	○
1 手术室注重护生手术室相关专科知识及专业技术的培养	○	○	○	○	○
2 临床带教教师有较强的专业能力	○	○	○	○	○
3 临床带教教师有较强的带教能力	○	○	○	○	○
4 临床带教教师能够有效指导护生,培养护生评判性思维能力和自主学习能力	○	○	○	○	○
5 在临床带教教师的指导下,护生有独立进行基本护理技术操作的机会	○	○	○	○	○
6 在临床带教教师的指导下,护生可以参与实践多种一二级简单手术类型的护理配合,如甲状腺手术、乳腺手术等	○	○	○	○	○
7 在临床带教教师的指导下,护生有机会学习观摩三四级护理疑难危重症手术的护理配合,如心血管手术、胸科手术等	○	○	○	○	○
8 护生有机会参与手术室护士专科培训、教学查房、学术交流会议等活动	○	○	○	○	○
6 在临床带教教师的指导下,护生可以参与实践多种一二级简单手术类型的护理配合,如甲状腺手术、乳腺手术等	○	○	○	○	○
7 在临床带教教师的指导下,护生有机会学习观摩三四级护理疑难危重症手术的护理配合,如心血管手术、胸科手术等	○	○	○	○	○
8 护生有机会参与手术室护士专科培训、教学查房、学术交流会议等活动	○	○	○	○	○
1 护生了解自己在手术室的角色和工作范畴	○	○	○	○	○
2 护生在手术室临床实习目标明确	○	○	○	○	○
3 护生的努力和进步能够得到赞赏和鼓励	○	○	○	○	○
4 护生不被安排做与手术室临床护理实习工作无关的事情	○	○	○	○	○
5 手术室对护生有全面的教学计划,实习工作安排合理有效	○	○	○	○	○
6 手术室的实习工作能够强化护生的专业知识与实践技能	○	○	○	○	○
7 手术室能保障护生在手术室的有效实习时间	○	○	○	○	○
8 护生的实习工作有专职人员(如护士长、教学组长等)负责管理	○	○	○	○	○
1 手术室的临床教学方式可以实现理论与实践相结合	○	○	○	○	○
2 采用先进的、创新的临床教学方法和考核形式提高实习效果	○	○	○	○	○
3 临床实习内容考虑护生个性化因素,因材施教,循序渐进,并根据临床实际情况灵活调整	○	○	○	○	○
4 鼓励护生在手术室临床实践中有创新思维,可以发现问题,在老师的指导下寻找解决方法	○	○	○	○	○
5 护生可以对手术室实习工作出现的问题提出合理化建议	○	○	○	○	○
6 护生之间有机会相互交流和学习	○	○	○	○	○
7 护生可以对手术室临床实习的体验进行评价和反馈	○	○	○	○	○

## Appendix

手术室护生临床学习环境评价.

说明:护理实习生,简称“护生”。请仔细阅读下面的题目,根据您的实习经历选出选择对应选项,回答没有对错之分。
